# Age-adjusted association of homologous recombination genes with ovarian cancer using clinical exomes as controls

**DOI:** 10.1186/s13053-019-0119-3

**Published:** 2019-07-15

**Authors:** Kevin J. Arvai, Maegan E. Roberts, Rebecca I. Torene, Lisa R. Susswein, Megan L. Marshall, Zhancheng Zhang, Natalie J. Carter, Lauren Yackowski, Erica S. Rinella, Rachel T. Klein, Kathleen S. Hruska, Kyle Retterer

**Affiliations:** 1 GeneDx, 207 Perry Pkwy, Gaithersburg, MD USA; 2 My Gene Team, Miami, Florida USA

**Keywords:** Ovarian Cancer, *BRIP1*, Exome sequencing

## Abstract

**Background:**

Genes in the homologous recombination pathway have shown varying results in the literature regarding ovarian cancer (OC) association. Recent case-control studies have used allele counts alone to quantify genetic associations with cancer.

**Methods:**

A retrospective case-control study was performed on 6,182 women with OC referred for hereditary cancer multi-gene panel testing (cases) and 4,690 mothers from trios who were referred for whole-exome sequencing (controls). We present age-adjusted odds ratios (OR_Adj_) to determine association of OC with pathogenic variants (PVs) in homologous recombination genes.

**Results:**

Significant associations with OC were observed in *BRCA1, BRCA2, RAD51C* and *RAD51D.* Other homologous recombination genes, *BARD1, NBN,* and *PALB2,* were not significantly associated with OC. *ATM* and *CHEK2* were only significantly associated with OC by crude odds ratio (OR_Crude_) or by OR_Adj_, respectively. However, there was no significant difference between OR_Crude_ and OR_Adj_ for these two genes. The significant association of PVs in *BRIP1* by OR_Crude_ (2.05, CI = 1.11 to 3.94, *P* = 0.03) was not observed by OR_Adj_ (0.87, CI = 0.41 to 1.93, *P* = 0.73). Interestingly, the confidence intervals of the two effect sizes were significantly different (*P* = 0.04).

**Conclusion:**

The lack of association of PVs in *BRIP1* with OC by OR_Adj_ is inconsistent with some previous literature and current management recommendations, highlighted by the significantly older age of OC onset for *BRIP1* PV carriers compared to non-carriers. By reporting OR_Adj_, this study presents associations that reflect more informed genetic contributions to OC when compared to traditional count-based methods.

**Electronic supplementary material:**

The online version of this article (10.1186/s13053-019-0119-3) contains supplementary material, which is available to authorized users.

## Background

Next-generation sequencing (NGS) has enabled clinical laboratories to analyze simultaneously a growing number of genes. Clinical multi-gene hereditary ovarian cancer (OC) panels include genes that function in the same homologous recombination (HR) DNA repair pathway as *BRCA1* and *BRCA2*, such as *ATM*, *BARD1*, *BRIP1*, *CHEK2*, *NBN*, *PALB2*, *RAD51C*, and *RAD51D*. These genes have been linked to hereditary ovarian cancer, but the extent to which some of these genes contribute to hereditary OC varies in the literature [1–7]. The identification of pathogenic or likely pathogenic variants (PVs) in these genes with ambiguous associations leads to difficult patient management decisions for clinicians and patients alike.

Identifying an appropriate control group for genetic association studies is vital for accurate estimation of cancer risks. Before high-throughput NGS, researchers relied on collaboration by pooling multi-center genotype data to achieve a large enough sample size to detect significant genetic associations [8, 9]. Recent association studies have drawn control data from the Exome Aggregation Consortium (ExAC), a public database which contains genotype information on over 60,000 individuals who have participated in genetic studies on conditions such as inflammatory bowel disease, heart disease, and schizophrenia [10–12]. The FLOSSIES database has also been used to perform genetic association studies specifically in women’s cancer [13]. The advantage of such cohorts are the large, readily available number of genotyped individuals with diverse ancestries and varied clinical histories, but drawbacks include the lack of specific phenotypic information including age and cancer history of the participants.

Clinical laboratories are addressing this limitation by leveraging data from exome sequencing (ES) tests performed internally [14]. In our laboratory, ES can be performed on trios (child and both parents) to help identify variants contributing to rare Mendelian diseases. Healthy parents from these trios serve as a reliable control cohort for hereditary cancer studies considering the breadth of genotyping coverage and the adequate phenotypic information provided. We performed a case-control study with an internal control population and report age-adjusted ORs to clarify equivocal genetic associations in HR genes with OC.

## Methods

### Cohort assembly

Genotypic and phenotypic data were collected from women who underwent germline genetic testing at GeneDx (Gaithersburg, MD) between 2013 and 2018. The study was conducted in accordance with all guidelines set forth by the Western Institutional Review Board, Puyallup, WA (WIRB 20162523). Informed consent for genetic testing was obtained from all individuals undergoing testing, and WIRB waived authorization for use of de-identified aggregate data for both cases and controls. Individuals or institutions who opted out of this type of data use were excluded. Cases were women with OC referred for *BRCA1*/*2* alone or multi-gene hereditary cancer panel testing who did not also have a targeted test for a known familial variant. Controls were mothers from a subset of complete trios referred for ES due to a neurodevelopmental delay in the proband. These mothers all self-reported that they did not have a disorder with genetic etiology. Both case and control cohorts were limited to those over 18 years of age and those who self-reported as White/Caucasian. Women were excluded if they were missing age at diagnosis or age at testing or if they had more than one PV, including homozygous variants, in any of the cancer predisposition genes available on ordered panels (Additional file 1: Table S1). Of all individuals referred for multi-gene hereditary cancer panel testing, 9,688 women were diagnosed with OC, 6,182 of whom met inclusion criteria. From the control cohort, there were 8,643 mothers of probands, of whom 4,690 satisfied the filtering criteria.

### Sequencing and variant calling

Cases were sequenced and genotyped with targeted NGS panels and a clinical bioinformatics pipeline as previously described [15]. Controls were sequenced by clinical ES as previously described using either Agilent SureSelect Human All Exon v4 or Agilent Clinical Research Exome capture protocols (Agilent, Santa Clara, CA) [16]. Separate from clinical ES testing, control samples were jointly genotyped across the entire cohort following Genome Analysis Toolkit Best Practices using HaplotypeCaller (version 3.7.0) in GVCF mode followed by GenotypeGVCFs and variant quality score recalibration [17–19]. Single nucleotide variants with genotype quality less than 50 and insertions and deletions (indels) with genotype quality less than 99 were considered low confidence, and therefore not included in the analysis. Additionally, for all novel prospective PVs in controls, de-identified NGS alignment data were manually inspected to remove suspicious variants. Sequencing methods for cases and controls were evaluated for potential bias (Additional file 2: Additional Sequencing Methods and Table S2). Copy number variants were not evaluated as part of this study.

### Variant classification

**Fig. 1:**
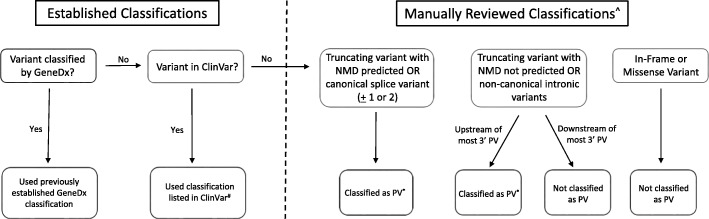
Rule-Based Algorithm Used to Classify Variants. ^#^No classification discrepancies were identified for variants falling into this category (not previously seen at GeneDx but in ClinVar). ^^^Variants requiring manual review were not classified based on strict ACMG criteria as is done for variants that were clinically reported. Variants were classified as either a PV, which includes variants that would meet criteria for a pathogenic or likely pathogenic classification, or not a PV. Variants determined to be not a PV were not worked up further to determine if they would be classified as variant of uncertain significance, likely benign, or benign. ^*^NGS data manually assessed to ensure variant was real. Default as real if it was determined that the variant in question could not be confidently called real or not real based on NGS data. NMD = nonsense mediated decay

Analysis of genetic variants were limited to those located in the HR genes, *ATM*, *BARD1*, *BRCA1*, *BRCA2*, *BRIP1*, *CHEK2*, *NBN*, *PALB2*, *RAD51C*, and *RAD51D*. For cases, the pathogenic classification of each variant was reviewed according to internal protocol, which follows ACMG/AMP guidelines [20]. Exon-level deletions and duplications were not included in the PV count since detection of these copy-number variants from ES is more limited and variable than from panel testing. Pathogenic status of variants in controls were systematically classified using a rule-based algorithm (Fig. 1). Variants that were previously classified in cases were assigned the same classification. Novel variants in the controls were assigned ClinVar classifications or were manually classified when ClinVar classifications were not available or conflicting. ClinVar classifications were obtained from the variant call format file released September 5, 2017 [21]. The common founder pathogenic missense variants, CHEK2*I157T and CHEK2*S428F were excluded from both cases and controls. All PVs are listed in Additional file 3: Table S3.

### Statistical analysis

A two-sample, independent t-test was performed to compare the mean age between cases and controls. Genetic associations were estimated by ORs using Firth bias-corrected logistic regression [22]. Crude ORs (OR_Crude_) were generated by simple logistic regression using status of PV in a gene as the independent variable. Multivariable logistic regression was performed with PV status and patient age (diagnosis in cases, at time of testing in controls) as independent variables to generate OR_Adj_. Reported 95% confidence intervals (CIs) were calculated using penalized profile likelihood. A z-test was performed to compare OR_Crude_ and OR_Adj_ for each gene (Additional file 4). The aggregate PV prevalence per-gene was compared to reported prevalence from Lilyquist et al. using Fisher’s exact test [23]. To correct for multiple independent tests, a Benjamini-Hochberg false discovery rate (FDR) correction was applied and a corrected *p*-value significance threshold of 0.05 was used when considering genetic association results and results from Fisher’s exact test. The t-test and z-tests were two-sided and *P* < 0.05 were considered significant. A sensitivity analysis was performed by reporting OC associations in a subset of women who were diagnosed with serous OC subtype (Additional file 5: Table S4). Finally, a power analysis was performed using a test of proportions to determine the statistical power of the study. Conditions of the test used the total cohort sizes, assumed a significance level of 0.05, and a subjective, but realistic, assumption that rate of PVs in each cohort were 0.01, and 0.005, respectively. Statistical analyses were performed using the R programming language.

## Results

### Cohort summary

After filtering into final cohorts, the mean age (standard deviation) at OC diagnosis was significantly higher in cases than age at testing in controls (58.8 (13.4) versus 41.9 (9.4), *P* < .05, t-test). The median age at diagnosis of PV carriers in OC cases ranged from 53 years in *BRCA1* to 67 years in *BRIP1*, while the median age of testing of PV carriers in controls ranged from 32.5 years in *RAD51D* to 46 years in *BRIP1* (Table 1). There were 780 (12.6%) OC cases who also had a breast cancer diagnosis. Across all genes, the observed aggregate PV prevalence in either the case or control cohort were not significantly different, respectively, from those reported by Lilyquist et al. (*P* = 0.05, Fisher’s exact test). There were 588 women of the 6,182 cases who harbored PVs (9.5%) while 98 of the 4,690 controls (2.1%) harbored PVs (Table 1).

**Table 1:** Associations of ovarian cancer with pathogenic variants in homologous recombination genes among self-reported white women

Gene	Median Age of PV Carriers (Cases^a^, Controls^b^)	Crude Odds Ratio (CI)	*p*-value, Crude^c^	Adjusted Odds Ratio (CI)	*p*-value, Adjusted^c^	Case Carriers, No. (Non-Carriers)^d^	Control Carriers, No. (Non-Carriers)	*p*-value, z-test^e^
*ATM*	55.0, 47.0	2.01. (1.15 to 3.64)	0.03	1.72 (0.89 to 3.44)	0.18	35 (4724)	17 (4673)	0.35
*BARD1*	60.0, 34.5	1.59 (0.31 to 9.56)	0.57	6.30 (0.55 to 74.25)	0.19	3 (4122)	2 (4688)	0.80
*BRCA1*	53.0, 37.0	38.46 (17.03 to 115.32)	1.03 × 10^−47^	47.80 (20.76 to 145.03)	1.87 × 10^−46^	219 (5944)	4 (4686)	0.60
*BRCA2*	63.0, 40.0	8.19 (5.22 to 13.67)	1.17 × 10^−28^	5.10 (3.12 to 8.83)	2.89 × 10^−12^	193 (5969)	18 (4672)	0.14
*BRIP1*	67.0, 46.0	2.05 (1.11 to 3.94)	0.03	0.87 (0.41 to 1.93)	0.73	30 (4805)	14 (4676)	0.04
*CHEK2*	52.0, 42.0	1.62 (1.00 to 2.69)	0.07	2.64 (1.48 to 4.79)	2.36 × 10^−3^	45 (4923)	24 (4287)	0.79
*NBN*	64.5, 37.0	0.52 (0.15 to 1.54)	0.27	0.45 (0.10 to 1.82)	0.33	4 (4232)	9 (4681)	0.46
*PALB2*	62.0, 39.0	2.06 (0.90 to 5.19)	0.11	1.78 (0.61 to 5.59)	0.33	16 (5004)	7 (4683)	0.50
*RAD51C*	61.0, 39.0	15.28 (3.97 to 137.01)	6.29 × 10^−06^	12.09 (2.78 to 114.43)	7.85 × 10^−04^	23 (4809)	1 (4689)	0.34
*RAD51D*	60.0, 32.5	7.99 (2.56 to 39.77)	1.97 × 10^−04^	8.38 (2.17 to 47.23)	2.51 × 10^−03^	20 (4809)	2 (4688)	0.44

^a^Age (in years) at time of ovarian cancer diagnosis

^b^Age (in years) at time of testing

^c^Corrected for False Discovery Rate

^d^All genes were not tested for every sample

^e^Comparing crude odds ratio to adjusted odds ratio

*CI* Confidence Interval, *No*. Number

### Genetic associations

Well-established OC susceptibility genes showed significant associations when measured by both OR_Crude_ and OR_Adj_: *BRCA1* (OR_Crude_ = 38.46, CI = 17.03 to 115.32, *P* = 1.03 × 10^− 47^; OR_Adj_ = 47.80, CI = 20.76 to 145.03, *P* = 1.87 × 10^− 46^)*, BRCA2* (OR_Crude_ = 8.19, CI = 5.22 to 13.67, *P* = 1.17 × 10^− 28^; OR_Adj_ = 5.10, CI = 3.12 to 8.83, *P* = 2.89 × 10^− 12^), *RAD51C* (OR_Crude_ = 15.28, CI =3.97 to 137.01, *P* = 6.09x^− 06^; OR_Adj_ = 12.09, CI = 2.78 to 114.43, *P* = 7.85 × 10^− 04^), and *RAD51D* (OR_Crude_ = 7.99, CI = 2.56 to 39.77, *P* = 1.97 × 10^− 04^; OR_Adj_ = 8.38, CI =2.17 to 47.23, *P* = 2.51 × 10^− 03^) (Table 1).

Some genes with no significant association with OC measured by OR_Crude_ consistently showed no significant association with OC as measured by OR_Adj_, including *BARD1* (OR_Crude_ = 1.59, CI = 0.31 to 9.56, *P* = 0.57; OR_Adj_ = 6.30, CI =0.55 to 74.25, *P* = 0.19), *NBN* (OR_Crude_ = 0.52, CI = 0.15 to 1.54, *P* = 0.27; OR_Adj_ = 0.45, CI = 0.10 to 1.82, *P* = 0.33), and *PALB2* (OR_Crude_ = 2.06, CI = 0.90 to 5.19, *P* = 0.11; OR_Adj_ = 1.78, CI = 0.61 to 5.59, P = 0.33).

*ATM* (OR_Crude_ = 2.01, CI = 1.15 to 3.64, *P* = 0.03; OR_Adj_ = 1.72, CI = 0.89 to 3.44, *P* = 0.18) and *BRIP1* (OR_Crude_ = 2.05, CI = 1.11 to 3.94, P = 0.03; OR_Adj_ = 0.87, CI =0.41 to 1.93, *P* = 0.73) were significantly associated with OC by OR_Crude_ but were not significantly associated with OC by OR_Adj_. Conversely, *CHEK2* (OR_Crude_ = 1.62, CI = 1.00 to 2.69, *P* = 0.07; OR_Adj_ = 2.64, CI = 1.48 to 4.79, *P* = 2.36 × 10^− 3^) was not significantly associated with OC by OR_Crude_ but was significantly associated with OC by OR_Adj_.

### Power analysis and effect size comparison

The power analysis revealed that the study can detect a difference in proportions from 0.01 to 0.005 with 85.9% power. The per-gene comparison of effect sizes was not significantly different for the majority of the genes evaluated. The single exception was *BRIP1*, for which OR_Adj_ was significantly lower than OR_Crude_ (z-test, *P* = 0.04) (Table 1).

## Discussion

Our approach is supported by the agreement of OR_Crude_ and OR_Adj_ with previously reported ORs for *BRCA1*, *BRCA2*, *RAD51C*, and *RAD51D*, genes with clearly established OC associations. The other HR pathway genes have been reported with conflicting evidence for association with OC [10, 14, 23–25]. Association of PVs in each these genes with OC are in agreement with at least one of these studies by either OR_Crude_ or OR_Adj_. While this validates that our methodology is comparable, it also highlights the disparity of concordance among these studies.

To check for potential ascertainment bias, PV prevalence in cases and controls was compared to those previously reported by Lilyquist et al. [26] Given the similar ascertainment of cases and robustness of the estimates from the ExAC non-Finnish European control population, this data serves as a germane baseline for comparison. The prevalence of PV was not significantly different in any gene (Additional file 6: Table S5). Small differences in PV counts due to differences in variant classification between studies can have a significant impact on effect size, especially for genes where PVs are rare. For example, Weber-Lassalle et al. reported 9 of 7,325 (0.12%) European PV carriers from the FLOSSIES database; however, our variant classification system would have reported 14 of 7,325 (0.19%), on the basis of including 5 European carriers of *BRIP1* c.139C > A (ClinVar: SCV000210833.12) (Fig. 1) [13].

Genes in which PVs are rare in one cohort (four or less PVs), *BARD1*, *BRCA1*, *NBN*, *RAD51C* and *RAD51D,* result in wide confidence intervals for the ORs. For example, *RAD51C* appears to confer greater risk than *BRCA2*, a gene whose association with OC has been well-established. However, with the exception of *CHEK2*, confidence intervals for ORs in all HR genes overlapped with those previously published, indicating the true effect size falls somewhere in the overlapping range [10]. Results from the power analysis can be interpreted that genes with moderate effect sizes are adequately powered to detect significant association but the result also supports the argument in favor of performing studies on genotyped cohorts large enough to sufficiently detect genetic associations in genes where PVs rare and the effect sizes are small.

No significant association with OC were consistently observed with PVs in *BARD1, NBN, and PALB2*. The reported low frequency of PVs in *BARD1*, similar to other studies, will require still larger sample sizes to detect a significant effect [10, 23, 24]. Association of *NBN* with OC is the low to moderate risk HR pathway gene with the most concordant results. It is widely regarded as not significantly associated with OC, with the exception of Lilyquist, et al. who reported that its association was “marginally significant” [23]. Additionally, Lilyquist et al. initially reported a significant association of *PALB2* with OC, but the association was lost upon removal of women with a personal or family history of breast cancer [23]. We also report no significant association of *PALB2* with OC.

Multiple studies have reported *ATM* as a moderate risk OC gene [14, 23]. The observed significance by OR_Crude_ is concordant with these results. In controls, *ATM* PV carriers had the highest median age (47 years, time of testing) of any HR pathway genes. Given that PVs in *ATM* were only moderately associated with OC by OR_Crude_, the median age of PV carriers was high enough compared to the median age of non-carriers to decrease the OR_Adj_ below the significance threshold. Notably, an insignificant z-test result indicates that the true effect size is likely to overlap the two reported CIs. The presence of undiagnosed cancer patients in the control cohort could falsely lower the calculated ORs, a similar effect was previously described in a study which used the ExAC controls with cancer samples included [10].

A well-known breast cancer risk gene, *CHEK2*, has been consistently reported to have no significant association with OC [10, 14, 23]. Similar to reported associations from *ATM*, the observed significance by OR_Crude_ is concordant with previously reported associations. In contrast, however, *CHEK2* PV carriers in cases had the lowest median age (52 years, time of OC diagnosis) of any of the HR pathway genes, which increased the OR_Adj_ enough to reach the level of significant association with OC. Again, similar to *ATM*, the z-test comparing the two effect sizes was insignificant, indicating that the true effect size is likely in the overlapping range of the CIs (Table 1). When limiting cases to women with serous OC pathology, association of OC with PVs in *CHEK2* disappears and the median age of OC diagnosis in PV carriers increases from 52 years to 63.5 years (Additional file 5: Table S4). None of the 12 *CHEK2* PV carriers with serous OC subtype were also diagnosed with breast cancer (not shown), suggesting that the significant OR_Adj_ result from the larger case cohort was a direct effect from the bias introduced by the younger *CHEK2* PV carriers who were also diagnosed with breast cancer.

Multiple publications have concluded that *BRIP1* is significantly associated with OC [10, 13, 23, 24]. While OR_Crude_ in *BRIP1* was significantly associated with OC, the OR_Adj_ showed no OC association. The significantly decreased effect size in *BRIP1* can be attributed to PV carriers displaying the oldest median age at time of OC diagnosis and the second oldest median age at time of testing among all of the genes (67y case PV carriers, 46y control PV carriers; Table). Advanced age of PV carriers in *BRIP1* is not a novel observation [10, 13, 23, 24]. *BRIP1* was the only gene in which OR_Adj_ was significantly different than OR_Crude_, which, given PV carriers are older compared to non-carriers, suggests that *BRIP1* PV carriers are more likely to be diagnosed with late-onset OC. Recent updates to National Comprehensive Cancer Network® (NCCN) guidelines for *BRIP1* PV carriers include consideration of risk-reducing salpingo-oophorectomy at 45–50 years of age [26]. The consistently reported older age at time of diagnosis and the observed lack of association by OR_Adj_ suggests caution before surgical intervention and the need for further studies of larger cohorts of older controls, as previously recommended [27].

A recent study reported no significant association of *BRIP1* with OC, but our results reveal a clinically relevant factor that offers insight which may have contributed to the observed lack of association. Age (mean [SD]) at time of OC diagnosis in cases (55.7y [14.1]) and age at time of testing in controls (39.7y [14.7]) were both younger than our cohorts, which could lead to under-counting *BRIP1* PV carriers. Finally, it has been suggested that *BRIP1*’s OC association may be restricted to high-grade serous epithelial ovarian cancer, but when cases were restricted to women diagnosed with high-grade serous OC histologic subtype, the OC association was consistent (Additional file 5: Table S4) [7].

By presenting OR_Adj_ in addition to OR_Crude_ this study allows for comparison of the two effect sizes. This comparison provides insight into the age difference between PV carriers and non-carriers and enables inference of early/late onset OC. Genes with PV carriers who are older than non-carriers demonstrate decreased OR_Adj_ compared to OR_Crude_ and conversely, genes with PV carriers who are younger than non-carriers have increased OR_Adj_ compared to OR_Crude_. Comparing the two effect sizes for each gene using a z-test revealed that controlling for age did not significantly change the OR, except in *BRIP1*. A significant z-test result suggests that *BRIP1* PV carriers are more likely to be diagnosed with late-onset OC (Table 1).

As a referral laboratory, clinical information was limited to that provided by ordering providers with submitted samples. With ES, the referrals are not routinely submitted for cancer-related testing and, therefore, cancer history may not have been included in the clinical histories by the ordering physician. In addition, because the mean age of controls was lower than the mean age of OC diagnosis, it cannot be ruled out that some of these women will ultimately be diagnosed with OC. The OC referral cases could be biased toward a higher risk than the general ovarian cancer population. Variant detection sensitivity filters for controls were conservatively chosen which may lead to under-reporting of PVs and thus overestimation of ORs. Another source of possible under-reporting of PVs in controls is due to the incomplete gene coverage in controls, although the minor difference would have negligible effect (Additional file 2: Additional Sequencing Methods and Table S2). Copy number variants would have likely contributed a small number of PVs to cases and controls. But since this type of variant was not evaluated it acts as a source of bias toward under-reporting PVs. As not all PVs in controls were orthogonally confirmed as they were in cases, it is possible that a small number of control PVs were sequencing artifacts. Targeted *BRCA1* and *BRCA2* testing tends to have a lower positive yield compared to panel testing with other HR genes, as previously described [28]. The overall pathogenic variant rate in OC (9.5%) is lower than published rates from other studies [10, 23]. This can be attributed 873 women in the case cohort who underwent testing for *BRCA1* and/or *BRCA2* only.

## Conclusions

We present age-adjusted genetic associations for PVs in HR genes with OC, leveraging an internal control cohort of women who self-report as White/Caucasian. Our study design and analysis provide more informed estimates of association compared to recently published OC associations by reporting both OR_Crude_ and OR_Adj_. These results are most relevant for *BRIP1* PV carriers, as our findings for this gene are disparate from recent literature and conflict with current management guidelines.

## Additional files

Additional file 1: Genes on GeneDx inherited cancer panels. (DOCX 13 kb)

Additional file 2: Additional sequencing methods and statistics. Details of GeneDx sequencing QC for comparison between cases and controls. (DOCX 13 kb)

Additional file 3: List of all pathogenic variants. The gene, cDNA, and protein change for each variant found in this study. (DOCX 57 kb)

Additional file 4: Calculation of the z-Statistic. The formula used to determine if crude odds ratios were significantly different than adjusted odds ratios. (DOCX 12 kb)

Additional file 5: Serous-subtype ovarian cancer sensitivity analysis. Results when limited to women with serous-subtype ovarian cancer. (DOCX 14 kb)

Additional file 6: Comparison of pathogenic variant prevalence with Lilyquist et al. Results from Fisher’s Exact Test comparing prevalence of pathogenic variants in homologous recombination genes to Lilyquist et al. (DOCX 14 kb)

## Data Availability

The datasets generated and/or analysed during the current study are not publicly available due to patient privacy restrictions but are available from the corresponding author on reasonable request.

## Abbreviations

ACMG: American college of medical genetics
AMP: Association for Molecular Pathology
CI: Confidence interval
ClinVar: Public database of reports of human variation
ES: Exome sequencing
ExAC: Exome aggregation consortium
FDR: False discovery rate
FLOSSIES: A sequencing database of women who are cancer free and over age 70
HR: Homologous recombination
NCCN: National comprehensive cancer network®
NGS: Next generation sequencing
OC: Ovarian cancer
OR: Odds ratio
OR_Adj_: Adjusted odds ratio
OR_Crude_: Crude odds ratio
PV: Pathogenic variant

## References

[1] Casadei S, Norquist BM, Walsh T, Stray SM, Mandell JB, Lee MK (2011). Contribution to familial breast Cancer of inherited mutations in the BRCA2-interacting protein PALB2. Cancer Res.

[2] Loveday C, Turnbull C, Ramsay E, Hughes D, Ruark E, Frankum JR (2011). Germline mutations in RAD51D confer susceptibility to ovarian cancer. Nat Genet.

[3] Loveday C, Turnbull C, Ruark E, Xicola RMM, Ramsay E, Hughes D (2012). Germline RAD51C mutations confer susceptibility to ovarian cancer. Nat Genet.

[4] Pennington KP, Walsh T, Harrell MI, Lee MK, Pennil CC, Rendi MH (2014). Germline and somatic mutations in homologous recombination genes predict platinum response and survival in ovarian, fallopian tube, and peritoneal carcinomas. Clin Cancer Res An Off J Am Assoc Cancer Res.

[5] Meindl A, Hellebrand H, Wiek C, Erven V, Wappenschmidt B, Niederacher D (2010). Germline mutations in breast and ovarian cancer pedigrees establish RAD51C as a human cancer susceptibility gene. Nat Genet.

[6] Rafnar T, Gudbjartsson DF, Sulem P, Jonasdottir A, Sigurdsson A, Jonasdottir A (2011). Mutations in BRIP1 confer high risk of ovarian cancer. Nat Genet.

[7] Walsh T, Casadei S, Lee MK, Pennil CC, Nord AS, Thornton AM (2011). Mutations in 12 genes for inherited ovarian, fallopian tube, and peritoneal carcinoma identified by massively parallel sequencing. Proc Natl Acad Sci U S A.

[8] Schildkraut JM, Risch N, Thompson WD (1989). Evaluating genetic association among ovarian, breast, and endometrial cancer: evidence for a breast/ovarian cancer relationship. Am J Hum Genet.

[9] Consortium CBCC-C (2004). CHEK2*1100delC and susceptibility to breast cancer: a collaborative analysis involving 10,860 breast cancer cases and 9,065 controls from 10 studies. Am J Hum Genet.

[10] Norquist BM, Harrell MI, Brady MF, Walsh T, Lee MK, Gulsuner S (2016). Inherited mutations in women with ovarian carcinoma. JAMA Oncol.

[11] Couch FJ, Shimelis H, Hu C, Hart SN, Polley EC, Na J (2017). Associations between cancer predisposition testing panel genes and breast cancer. JAMA Oncol.

[12] Castéra L, Harter V, Muller E, Krieger S, Goardon N, Ricou A, et al. Landscape of pathogenic variations in a panel of 34 genes and cancer risk estimation from 5131 HBOC families. Genet Med. 2018;1 Available from: http://www.nature.com/articles/s41436-018-0005-9. Cited 2018 Aug 31. 10.1038/s41436-018-0005-9 29988077

[13] Weber-Lassalle N, Hauke J, Ramser J, Richters L, Groß E, Blümcke B (2018). BRIP1 loss-of-function mutations confer high risk for familial ovarian cancer, but not familial breast cancer. Breast Cancer Res.

[14] Lu HM, Li S, Black MH, Lee S, Hoiness R, Wu S (2019). Association of Breast and Ovarian Cancers with predisposition genes identified by large-scale sequencing. JAMA Oncol.

[15] Roberts ME, Jackson SA, Susswein LR, Zeinomar N, Ma X, Marshall ML (2018). MSH6 and PMS2 germ-line pathogenic variants implicated in Lynch syndrome are associated with breast cancer. Genet Med.

[16] Retterer K, Juusola J, Cho MT, Vitazka P, Millan F, Gibellini F (2016). Clinical application of whole-exome sequencing across clinical indications. Genet Med.

[17] Depristo MA, Banks E, Poplin R, Garimella KV, Maguire JR, Hartl C (2011). A framework for variation discovery and genotyping using next-generation DNA sequencing data. Nat Genet.

[18] Van der Auwera GA, Carneiro MO, Hartl C, Poplin R, del Angel G, Levy-Moonshine A (2013). From fastQ data to high-confidence variant calls: the genome analysis toolkit best practices pipeline. Curr Protoc Bioinformatics.

[19] Poplin R, Ruano-Rubio V, DePristo MA, Fennell TJ, Carneiro MO, Van der Auwera GA, et al. Scaling accurate genetic variant discovery to tens of thousands of samples. BioRxiv. 2017;201178 Available from: https://www.biorxiv.org/content/early/2017/11/14/201178.1. Cited 2018 Jan 29.

[20] Richards S, Aziz N, Bale S, Bick D, Das S, Gastier-Foster J (2015). Standards and guidelines for the interpretation of sequence variants: a joint consensus recommendation of the American college of medical genetics and genomics and the association for molecular pathology. Genet Med.

[21] Landrum MJ, Lee JM, Benson M, Brown GR, Chao C, Chitipiralla S (2018). ClinVar: improving access to variant interpretations and supporting evidence. Nucleic Acids Res.

[22] Firth D (1993). Bias Reduction of Maximum Likelihood Estimates. Biometrika.

[23] Lilyquist J, LaDuca H, Polley E, Davis BT, Shimelis H, Hu C (2017). Frequency of mutations in a large series of clinically ascertained ovarian cancer cases tested on multi-gene panels compared to reference controls. Gynecol Oncol.

[24] Ramus SJ, Song H, Dicks E, Tyrer JP, Rosenthal AN, Intermaggio MP, et al. Germline mutations in the BRIP1, BARD1, PALB2, and NBN genes in women with ovarian cancer. J Natl Cancer Inst. 2015;107(11) Available from: http://www.ncbi.nlm.nih.gov/pubmed/26315354. Cited 2018 Jun 27. 10.1093/jnci/djv214 PMC4643629 26315354

[25] Southey MC, Goldgar DE, Winqvist R, Pylkäs K, Couch F, Tischkowitz M (2016). PALB2, CHEK2 and ATM rare variants and cancer risk: data from COGS. J Med Genet.

[26] National Comprehensive Cancer Network (2017). Genetic/Familial High-Risk Assessment: Breast and Ovarian (Version I.2017).

[27] Balmaña J, Domchek SM (2015). BRIP1 as an ovarian cancer susceptibility gene: ready for the clinic?. J Natl Cancer Inst.

[28] Carter NJ, Marshall ML, Susswein LR, Zorn KK, Hiraki S, Arvai KJ (2018). Germline pathogenic variants identified in women with ovarian tumors. Gynecol Oncol.

