# Supplementary material for: Age-adjusted association of homologous recombination genes with ovarian cancer using clinical exomes as controls
Source: Hered Cancer Clin Pract. 2019 Jul 15;17:19. doi: 10.1186/s13053-019-0119-3 (PMC6631909; doi:10.1186/s13053-019-0119-3)
Supplement: Supplementary file 1 — Genes on GeneDx inherited cancer panels. (DOCX 13 kb) [file 13053_2019_119_MOESM1_ESM.docx]

**Additional file 1: Table S1.** Genes on GeneDx Inherited Cancer Panels.

| Gene |
| --- |
| *ALK* |
| *APC* |
| *ATM* |
| *AXIN2* |
| *BAP1* |
| *BARD1* |
| *BMPR1A* |
| *BRCA1* |
| *BRCA2* |
| *BRIP1* |
| *CDC73* |
| *CDH1* |
| *CDK4* |
| *CDKN2A* |
| *CHEK2* |
| *DICER1* |
| *EPCAM* |
| *FANCC* |
| *FH* |
| *FLCN* |
| *HOXB13* |
| *MAX* |
| *MEN1* |
| *MET* |
| *MITF* |
| *MLH1* |
| *MSH2* |
| *MSH6* |
| *MUTYH* |
| *NBN* |
| *NF1* |
| *NF2* |
| *NTHL1* |
| *PALB2* |
| *PHOX2B* |
| *PMS2* |
| *POLD1* |
| *POLE* |
| *POT1* |
| *PRKAR1A* |
| *PTCH1* |
| *PTEN* |
| *RAD51C* |
| *RAD51D* |
| *RB1* |
| *RECQL* |
| *RET* |
| *SCG5/GREM1* |
| *SDHA* |
| *SDHAF2* |
| *SDHB* |
| *SDHC* |
| *SDHD* |
| *SMAD4* |
| *SMARCA4* |
| *SMARCB1* |
| *STK11* |
| *SUFU* |
| *TMEM127* |
| *TP53* |
| *TSC1* |
| *TSC2* |
| *VHL* |
| *WT1* |
