# Supplementary material for: Age-adjusted association of homologous recombination genes with ovarian cancer using clinical exomes as controls
Source: Hered Cancer Clin Pract. 2019 Jul 15;17:19. doi: 10.1186/s13053-019-0119-3 (PMC6631909; doi:10.1186/s13053-019-0119-3)
Supplement: Supplementary file 2 — Additional sequencing methods and statistics. Details of GeneDx sequencing QC for comparison between cases and controls. (DOCX 13 kb) [file 13053_2019_119_MOESM2_ESM.docx]

**Additional Sequencing Methods Details**

To ensure consistency between the targeted gene regions, regions of interest (ROI), in cases and controls, analysis was limited to variants from coding exons plus twenty base pairs of adjacent intronic regions. Case ROIs were all sequenced to a depth of at least 15x and any gaps were filled in with targeted Sanger sequencing. In controls, *CHEK2* was notably not well covered in the SureSelect v4 capture with approximately one-third of ROIs not covered. These SureSelect v4 samples were thus excluded from our analysis for *CHEK2*. For the remaining set of controls all ROIs were sequenced to a mean depth of at least 15x in 99.5% (Q1 99.2%, Q3 99.5%) of sample targets across all genes.

**Additional file 2: Table S2.** Sequencing Statistics for Joint Genotyped Control Samples

| **Kit** | **N samples** |  |
| --- | --- | --- |
| Agilent SureSelect v4 | 1611 |  |
| Agilent Clinical Research Exome | 3956 |  |
|  |  |  |
| **Stats for Per Exon Coverage for Coding Exons for Each Gene Across All Control Samples** | | |
| **Gene** | **Median (Q1, Q3)** | **>=15x Coverage** |
| *ATM* | 103 (70 - 150) | 99.5% |
| *BARD1* | 100 (57 - 148) | 99.5% |
| *BRCA1* | 110 (65 - 184) | 97.7% |
| *BRCA2* | 110 (72 - 156) | 98.8% |
| *BRIP1* | 116 (75 -179) | 99.5% |
| *CHEK2^* | 149 (73 - 220) | 99.5% |
| *NBN* | 93.8 (57 - 152) | 95.9% |
| *PALB2* | 130 (84 -183) | 99.5% |
| *RAD51C* | 117 (79 - 169) | 99.7% |
| *RAD51D* | 126 (81 - 195) | 99.5% |

^excluding Agilent SureSelect v4

Coverage performance was comparable between both sequencing kits with an average increase in coverage on the Clinical Research Exome of <1%.
