# Supplementary material for: Age-adjusted association of homologous recombination genes with ovarian cancer using clinical exomes as controls
Source: Hered Cancer Clin Pract. 2019 Jul 15;17:19. doi: 10.1186/s13053-019-0119-3 (PMC6631909; doi:10.1186/s13053-019-0119-3)
Supplement: Supplementary file 3 — List of all pathogenic variants. The gene, cDNA, and protein change for each variant found in this study. (DOCX 57 kb) [file 13053_2019_119_MOESM3_ESM.docx]

**Additional file 3: Table S3.** List of all Pathogenic and Likely Pathogenic Variants.

| ***Gene*** | ***cDNA*** | ***Protein*** |
| --- | --- | --- |
| *ATM* | c.2T>C | p.Met1? |
| *ATM* | c.331+5G>A |  |
| *ATM* | c.378delT | p.Asp126Glufs*3 |
| *ATM* | c.622A>T | p.Lys208Ter |
| *ATM* | c.875C>T | p.Pro292Leu |
| *ATM* | c.1024_1027delAAAG | p.Glu343Ilefs*2 |
| *ATM* | c.1139_1142dupACAG | p.Ser381Argfs*27 |
| *ATM* | c.1339C>T | p.Arg447Ter |
| *ATM* | c.1402_1403delAA | p.Lys468Glufs*18 |
| *ATM* | c.1523delT | p.Gly509Glufs*3 |
| *ATM* | c.1561_1562delAG | p.Glu522Ilefs*43 |
| *ATM* | c.1898+1G>T |  |
| *ATM* | c.2921+1G>C |  |
| *ATM* | c.3130_3131insG | p.Asn1044Argfs*4 |
| *ATM* | c.3754_3756delTATinsCA | p.Tyr1252Glnfs*4 |
| *ATM* | c.3802delG | p.Val1268Ter |
| *ATM* | c.3848T>C | p.Leu1283Pro |
| *ATM* | c.4236+1G>T |  |
| *ATM* | c.5290delC | p.Leu1764Tyrfs*12 |
| *ATM* | c.5496+1G>T |  |
| *ATM* | c.5549delT | p.Leu1850Tyrfs*67 |
| *ATM* | c.5623C>T | p.Arg1875Ter |
| *ATM* | c.5712dupA | p.Ser1905Ilefs*25 |
| *ATM* | c.5932G>T | p.Glu1978Ter |
| *ATM* | c.6095G>A | p.Arg2032Lys |
| *ATM* | c.6100C>T | p.Arg2034Ter |
| *ATM* | c.6403_6404insTT | p.Arg2136Ter |
| *ATM* | c.6514delA | p.Thr2172Hisfs*63 |
| *ATM* | c.6679C>T | p.Arg2227Cys |
| *ATM* | c.6866_6867delCT | p.Ser2289Ter |
| *ATM* | c.6914_6915delAG | p.Leu2307Cysfs*65 |
| *ATM* | c.6997dupA | p.Thr2333Asnfs*40 |
| *ATM* | c.7271T>G | p.Val2424Gly |
| *ATM* | c.7327C>T | p.Arg2443Ter |
| *ATM* | c.7638_7646delTAGAATTTC | p.Arg2547_Ser2549del |
| *ATM* | c.7788G>A | p.Glu2596= |
| *ATM* | c.8147T>C | p.Val2716Ala |
| *ATM* | c.8266A>T | p.Lys2756Ter |
| *ATM* | c.8305delT | p.Trp2769Glyfs*37 |
| *ATM* | c.8395_8404del10 | p.Phe2799Lysfs*4 |
| *ATM* | c.8418+1_8418+4delGTGA |  |
| *ATM* | c.8833_8834delCT | p.Leu2945Valfs*10 |
| *ATM* | c.8977C>T | p.Arg2993Ter |
| *BARD1* | c.159-1G>T |  |
| *BARD1* | c.625_626insG | p.Lys209Argfs*5 |
| *BARD1* | c.1212C>G | p.Tyr404Ter |
| *BARD1* | c.1935_1954dup20 | p.Glu652Valfs*69 |
| *BRCA1* | c.68_69delAG | p.Glu23Valfs*17 |
| *BRCA1* | c.135-1G>A |  |
| *BRCA1* | c.143delT | p.Met48Serfs*2 |
| *BRCA1* | c.181T>G | p.Cys61Gly |
| *BRCA1* | c.212+1G>T |  |
| *BRCA1* | c.213-11T>G |  |
| *BRCA1* | c.310dupA | p.Ser104Lysfs*3 |
| *BRCA1* | c.329dupA | p.Glu111Glyfs*3 |
| *BRCA1* | c.427G>T | p.Glu143Ter |
| *BRCA1* | c.514delC | p.Gln172Asnfs*62 |
| *BRCA1* | c.547+1G>T |  |
| *BRCA1* | c.676delT | p.Cys226Valfs*8 |
| *BRCA1* | c.843_846delCTCA | p.Ser282Tyrfs*15 |
| *BRCA1* | c.850C>T | p.Gln284Ter |
| *BRCA1* | c.923delG | p.Ser308Thrfs*6 |
| *BRCA1* | c.930delG | p.Gln310Hisfs*4 |
| *BRCA1* | c.962G>A | p.Trp321Ter |
| *BRCA1* | c.1016dupA | p.Val340Glyfs*6 |
| *BRCA1* | c.1016delA | p.Lys339Argfs*2 |
| *BRCA1* | c.1059G>A | p.Trp353Ter |
| *BRCA1* | c.1175_1214del40 | p.Leu392Glnfs*5 |
| *BRCA1* | c.1277delC | p.Ser426Ter |
| *BRCA1* | c.1323_1324delAT | p.Ile441Metfs*2 |
| *BRCA1* | c.1329_1330delAA | p.Ser444Ter |
| *BRCA1* | c.1335_1336delAA | p.Arg446Serfs*9 |
| *BRCA1* | c.1360_1361delAG | p.Ser454Ter |
| *BRCA1* | c.1504_1508delTTAAA | p.Leu502Alafs*2 |
| *BRCA1* | c.1556delA | p.Lys519Argfs*13 |
| *BRCA1* | c.1600C>T | p.Gln534Ter |
| *BRCA1* | c.1601_1602delAG | p.Gln534Argfs*3 |
| *BRCA1* | c.1687C>T | p.Gln563Ter |
| *BRCA1* | c.1695dupG | p.Lys566Glufs*4 |
| *BRCA1* | c.1760dupT | p.Ser588Lysfs*20 |
| *BRCA1* | c.1881_1884delCAGT | p.Ser628Glufs*3 |
| *BRCA1* | c.1916T>A | p.Leu639Ter |
| *BRCA1* | c.1923dupT | p.Asp642Ter |
| *BRCA1* | c.1953_1956delGAAA | p.Lys653Serfs*47 |
| *BRCA1* | c.1961delA | p.Lys654Serfs*47 |
| *BRCA1* | c.2019delA | p.Glu673Aspfs*28 |
| *BRCA1* | c.2035A>T | p.Lys679Ter |
| *BRCA1* | c.2125_2126insA | p.Phe709Tyrfs*3 |
| *BRCA1* | c.2309C>A | p.Ser770Ter |
| *BRCA1* | c.2338C>T | p.Gln780Ter |
| *BRCA1* | c.2411_2412delAG | p.Gln804Leufs*5 |
| *BRCA1* | c.2457delC | p.Asp821Ilefs*25 |
| *BRCA1* | c.2467delA | p.Arg823Glufs*23 |
| *BRCA1* | c.2475delC | p.Asp825Glufs*21 |
| *BRCA1* | c.2679_2682delGAAA | p.Lys893Asnfs*106 |
| *BRCA1* | c.2681_2682delAA | p.Lys894Thrfs*8 |
| *BRCA1* | c.2694dupA | p.Val899Serfs*4 |
| *BRCA1* | c.2722G>T | p.Glu908Ter |
| *BRCA1* | c.2783delG | p.Gly928Alafs*72 |
| *BRCA1* | c.2834_2836delGTAinsC | p.Ser945Thrfs*6 |
| *BRCA1* | c.2934T>G | p.Tyr978Ter |
| *BRCA1* | c.2959A>T | p.Lys987Ter |
| *BRCA1* | c.2983A>T | p.Lys995Ter |
| *BRCA1* | c.3048_3052dupTGAGA | p.Asn1018Metfs*8 |
| *BRCA1* | c.3084_3094del11 | p.Asn1029Argfs*5 |
| *BRCA1* | c.3178G>T | p.Glu1060Ter |
| *BRCA1* | c.3228_3229delAG | p.Gly1077Alafs*8 |
| *BRCA1* | c.3285delA | p.Lys1095Asnfs*14 |
| *BRCA1* | c.3307_3308insC | p.Cys1103Serfs*2 |
| *BRCA1* | c.3331_3334delCAAG | p.Gln1111Asnfs*5 |
| *BRCA1* | c.3403C>T | p.Gln1135Ter |
| *BRCA1* | c.3481_3491del11 | p.Glu1161Phefs*3 |
| *BRCA1* | c.3485delA | p.Asp1162Valfs*48 |
| *BRCA1* | c.3514G>T | p.Glu1172Ter |
| *BRCA1* | c.3526delG | p.Val1176Phefs*34 |
| *BRCA1* | c.3607C>T | p.Arg1203Ter |
| *BRCA1* | c.3627dupA | p.Glu1210Argfs*9 |
| *BRCA1* | c.3642_3643delGA | p.Asn1215Leufs*3 |
| *BRCA1* | c.3668_3671dupTTCC | p.Cys1225Serfs*10 |
| *BRCA1* | c.3672delC | p.Cys1225Alafs*10 |
| *BRCA1* | c.3700_3704delGTAAA | p.Val1234Glnfs*8 |
| *BRCA1* | c.3748G>T | p.Glu1250Ter |
| *BRCA1* | c.3756_3759delGTCT | p.Ser1253Argfs*10 |
| *BRCA1* | c.3770_3771delAG | p.Glu1257Glyfs*9 |
| *BRCA1* | c.3869_3870delAA | p.Lys1290Metfs*4 |
| *BRCA1* | c.3937C>T | p.Gln1313Ter |
| *BRCA1* | c.3967delC | p.Gln1323Lysfs*2 |
| *BRCA1* | c.4035delA | p.Glu1346Lysfs*20 |
| *BRCA1* | c.4065_4068delTCAA | p.Asn1355Lysfs*10 |
| *BRCA1* | c.4096+1G>A |  |
| *BRCA1* | c.4137delT | p.Glu1380Lysfs*13 |
| *BRCA1* | c.4182_4183dupTC | p.Gln1395Leufs*11 |
| *BRCA1* | c.4183C>T | p.Gln1395Ter |
| *BRCA1* | c.4327C>T | p.Arg1443Ter |
| *BRCA1* | c.4391_4393delCTAinsTT | p.Pro1464Leufs*2 |
| *BRCA1* | c.4484G>C | p.Arg1495Thr |
| *BRCA1* | c.4484G>T | p.Arg1495Met |
| *BRCA1* | c.4485-1G>A |  |
| *BRCA1* | c.4675+1G>A |  |
| *BRCA1* | c.4689C>G | p.Tyr1563Ter |
| *BRCA1* | c.4868C>G | p.Ala1623Gly |
| *BRCA1* | c.4986+3G>C |  |
| *BRCA1* | c.5074G>C | p.Asp1692His |
| *BRCA1* | c.5095C>T | p.Arg1699Trp |
| *BRCA1* | c.5096G>A | p.Arg1699Gln |
| *BRCA1* | c.5109T>G | p.Tyr1703Ter |
| *BRCA1* | c.5123C>A | p.Ala1708Glu |
| *BRCA1* | c.5136G>A | p.Trp1712Ter |
| *BRCA1* | c.5193+1G>T |  |
| *BRCA1* | c.5207T>C | p.Val1736Ala |
| *BRCA1* | c.5215_5216delGA | p.Asp1739Cysfs*90 |
| *BRCA1* | c.5266dupC | p.Gln1756Profs*74 |
| *BRCA1* | c.5277+1G>A |  |
| *BRCA1* | c.5363G>T | p.Gly1788Val |
| *BRCA1* | c.5406+4A>G |  |
| *BRCA1* | c.5503C>T | p.Arg1835Ter |
| *BRCA2* | c.170dupA | p.Tyr57Ter |
| *BRCA2* | c.349_350delCT | p.Leu117Serfs*6 |
| *BRCA2* | c.444T>A | p.Cys148Ter |
| *BRCA2* | c.475G>A | p.Val159Met |
| *BRCA2* | c.631+2T>G |  |
| *BRCA2* | c.658_659delGT | p.Val220Ilefs*4 |
| *BRCA2* | c.891_899delAACAGTTGTins10 | p.Thr298Ilefs*7 |
| *BRCA2* | c.1029delA | p.Lys343Asnfs*6 |
| *BRCA2* | c.1114_1115delAAinsC | p.Asn372Leufs*27 |
| *BRCA2* | c.1189_1190insTTAG | p.Gln397Leufs*25 |
| *BRCA2* | c.1265delA | p.Asn422Ilefs*8 |
| *BRCA2* | c.1296_1297delGA | p.Asn433Glnfs*18 |
| *BRCA2* | c.1310_1313delAAGA | p.Lys437Ilefs*22 |
| *BRCA2* | c.1389_1390delAG | p.Val464Glyfs*3 |
| *BRCA2* | c.1411G>T | p.Glu471Ter |
| *BRCA2* | c.1440C>A | p.Cys480Ter |
| *BRCA2* | c.1670T>G | p.Leu557Ter |
| *BRCA2* | c.1754delA | p.Lys585Argfs*29 |
| *BRCA2* | c.1813dupA | p.Ile605Asnfs*11 |
| *BRCA2* | c.1929delG | p.Arg645Glufs*15 |
| *BRCA2* | c.2330dupA | p.Asp777Glufs*11 |
| *BRCA2* | c.2339C>G | p.Ser780Ter |
| *BRCA2* | c.2653_2656delGACA | p.Asp885Metfs*9 |
| *BRCA2* | c.2808_2811delACAA | p.Ala938Profs*21 |
| *BRCA2* | c.2830A>T | p.Lys944Ter |
| *BRCA2* | c.2855C>A | p.Ala952Glu |
| *BRCA2* | c.2957dupA | p.Asn986Lysfs*2 |
| *BRCA2* | c.3059_3060delCT | p.Ser1020Ter |
| *BRCA2* | c.3103G>T | p.Glu1035Ter |
| *BRCA2* | c.3158T>G | p.Leu1053Ter |
| *BRCA2* | c.3272T>A | p.Leu1091Ter |
| *BRCA2* | c.3545_3546delTT | p.Phe1182Ter |
| *BRCA2* | c.3599_3600delGT | p.Cys1200Ter |
| *BRCA2* | c.3641dupT | p.Phe1216Valfs*2 |
| *BRCA2* | c.3680_3681delTG | p.Leu1227Glnfs*5 |
| *BRCA2* | c.3689delC | p.Ser1230Leufs*9 |
| *BRCA2* | c.3708dupA | p.Ala1237Serfs*6 |
| *BRCA2* | c.3744_3747delTGAG | p.Ser1248Argfs*10 |
| *BRCA2* | c.3847_3848delGT | p.Val1283Lysfs*2 |
| *BRCA2* | c.4228dupA | p.Thr1410Asnfs*4 |
| *BRCA2* | c.4284dupT | p.Gln1429Serfs*9 |
| *BRCA2* | c.4339delG | p.Val1447Ter |
| *BRCA2* | c.4398_4402delACATT | p.Leu1466Phefs*2 |
| *BRCA2* | c.4415_4418delAGAA | p.Lys1472Thrfs*6 |
| *BRCA2* | c.4449delA | p.Asp1484Thrfs*2 |
| *BRCA2* | c.4478_4481delAAAG | p.Glu1493Valfs*10 |
| *BRCA2* | c.4638delT | p.Phe1546Leufs*22 |
| *BRCA2* | c.4677delT | p.Phe1559Leufs*9 |
| *BRCA2* | c.4808dupA | p.Asn1603Lysfs*6 |
| *BRCA2* | c.4876_4877delAA | p.Asn1626Serfs*12 |
| *BRCA2* | c.4889C>G | p.Ser1630Ter |
| *BRCA2* | c.4936_4939delGAAA | p.Glu1646Glnfs*23 |
| *BRCA2* | c.4947_4948delAA | p.Pro1651Cysfs*14 |
| *BRCA2* | c.4965C>G | p.Tyr1655Ter |
| *BRCA2* | c.5065_5066delGCinsAAA | p.Ala1689Lysfs*6 |
| *BRCA2* | c.5073dupA | p.Trp1692Metfs*3 |
| *BRCA2* | c.5106_5109delAGAA | p.Arg1704Ter |
| *BRCA2* | c.5110A>T | p.Arg1704Ter |
| *BRCA2* | c.5217_5220delTTTA | p.Tyr1739Ter |
| *BRCA2* | c.5350_5351delAA | p.Asn1784Hisfs*2 |
| *BRCA2* | c.5434G>T | p.Glu1812Ter |
| *BRCA2* | c.5471dupA | p.Asn1824Lysfs*5 |
| *BRCA2* | c.5576_5579delTTAA | p.Ile1859Lysfs*3 |
| *BRCA2* | c.5609_5610delTCinsAG | p.Phe1870Ter |
| *BRCA2* | c.5645C>A | p.Ser1882Ter |
| *BRCA2* | c.5681dupA | p.Tyr1894Ter |
| *BRCA2* | c.5722_5723delCT | p.Leu1908Argfs*2 |
| *BRCA2* | c.5828delC | p.Ser1943Leufs*20 |
| *BRCA2* | c.5857G>T | p.Glu1953Ter |
| *BRCA2* | c.5864C>A | p.Ser1955Ter |
| *BRCA2* | c.5946delT | p.Ser1982Argfs*22 |
| *BRCA2* | c.5991_5995delACAAG | p.Arg1997Serfs*4 |
| *BRCA2* | c.6129dupA | p.Gly2044Argfs*5 |
| *BRCA2* | c.6267_6269delGCAinsC | p.Glu2089Aspfs*2 |
| *BRCA2* | c.6275_6276delTT | p.Leu2092Profs*7 |
| *BRCA2* | c.6373delA | p.Thr2125Profs*12 |
| *BRCA2* | c.6444dupT | p.Ile2149Tyrfs*2 |
| *BRCA2* | c.6468_6469delTC | p.Gln2157Ilefs*18 |
| *BRCA2* | c.6486_6489delACAA | p.Lys2162Asnfs*5 |
| *BRCA2* | c.6591_6592delTG | p.Glu2198Asnfs*4 |
| *BRCA2* | c.6662delA | p.Asn2221Thrfs*8 |
| *BRCA2* | c.6833_6837delTCTTA | p.Ile2278Serfs*13 |
| *BRCA2* | c.6959T>A | p.Leu2320Ter |
| *BRCA2* | c.6980delT | p.Leu2327Ter |
| *BRCA2* | c.7007G>A | p.Arg2336His |
| *BRCA2* | c.7007G>C | p.Arg2336Pro |
| *BRCA2* | c.7069_7070delCT | p.Leu2357Valfs*2 |
| *BRCA2* | c.7147dupT | p.Tyr2383Leufs*9 |
| *BRCA2* | c.7251_7252delCA | p.His2417Glnfs*3 |
| *BRCA2* | c.7388_7389insGTTAATAT | p.Asn2463Lysfs*9 |
| *BRCA2* | c.7558C>T | p.Arg2520Ter |
| *BRCA2* | c.7618-1G>A |  |
| *BRCA2* | c.7679_7680delTT | p.Phe2560Serfs*5 |
| *BRCA2* | c.7876T>C | p.Trp2626Arg |
| *BRCA2* | c.7878G>C | p.Trp2626Cys |
| *BRCA2* | c.7946delC | p.Pro2649Glnfs*8 |
| *BRCA2* | c.7976+1G>A |  |
| *BRCA2* | c.7976G>A | p.Arg2659Lys |
| *BRCA2* | c.8167G>C | p.Asp2723His |
| *BRCA2* | c.8243G>A | p.Gly2748Asp |
| *BRCA2* | c.8247_8248delGA | p.Lys2750Aspfs*13 |
| *BRCA2* | c.8331+2T>C |  |
| *BRCA2* | c.8488-1G>A |  |
| *BRCA2* | c.8537_8538delAG | p.Glu2846Glyfs*22 |
| *BRCA2* | c.8575delC | p.Gln2859Lysfs*4 |
| *BRCA2* | c.8754+4A>G |  |
| *BRCA2* | c.8904delC | p.Val2969Cysfs*7 |
| *BRCA2* | c.8954-1_8955delGTTinsAA |  |
| *BRCA2* | c.8975_9100del126 | p.Pro2992_Thr3033del |
| *BRCA2* | c.9004G>A | p.Glu3002Lys |
| *BRCA2* | c.9117G>A | p.Pro3039= |
| *BRCA2* | c.9118-2A>G |  |
| *BRCA2* | c.9196C>T | p.Gln3066Ter |
| *BRCA2* | c.9227G>A | p.Gly3076Glu |
| *BRCA2* | c.9253dupA | p.Thr3085Asnfs*26 |
| *BRCA2* | c.9382C>T | p.Arg3128Ter |
| *BRCA2* | c.9403delC | p.Leu3135Phefs*28 |
| *BRCA2* | c.9513_9516delACTT | p.Leu3172Alafs*44 |
| *BRCA2* | c.9580_9581delCC | p.Pro3194Asnfs*2 |
| *BRCA2* | c.9599C>G | p.Ser3200Ter |
| *BRCA2* | c.9883C>T | p.Gln3295Ter |
| *BRIP1* | c.1_2delAT | p.Met1? |
| *BRIP1* | c.290_293delACAA | p.Asn97Metfs*3 |
| *BRIP1* | c.627+1G>A |  |
| *BRIP1* | c.1045G>C | p.Ala349Pro |
| *BRIP1* | c.1201_1204dupTGTG | p.Ala402Valfs*21 |
| *BRIP1* | c.1372G>T | p.Glu458Ter |
| *BRIP1* | c.1758delT | p.His587Metfs*3 |
| *BRIP1* | c.1871C>A | p.Ser624Ter |
| *BRIP1* | c.2011_2012insT | p.Glu671Valfs*5 |
| *BRIP1* | c.2038_2039dupTT | p.Leu680Phefs*9 |
| *BRIP1* | c.2108delAinsTCC | p.Lys703Ilefs*3 |
| *BRIP1* | c.2109_2110insCC | p.Leu704Profs*2 |
| *BRIP1* | c.2255_2256delAA | p.Lys752Argfs*12 |
| *BRIP1* | c.2258-1G>A |  |
| *BRIP1* | c.2392C>T | p.Arg798Ter |
| *BRIP1* | c.2400C>G | p.Tyr800Ter |
| *BRIP1* | c.2492+2dupT |  |
| *BRIP1* | c.2684_2687delCCAT | p.Ser895Ter |
| *BRIP1* | c.2765T>G | p.Leu922Ter |
| *BRIP1* | c.2992_2995delAAGA | p.Lys998Glufs*60 |
| *BRIP1* | c.3016_3017insT | p.Asn1006Ilefs*10 |
| *CHEK2* | c.3G>A | p.Met1? |
| *CHEK2* | c.190G>A | p.Glu64Lys |
| *CHEK2* | c.349A>G | p.Arg117Gly |
| *CHEK2* | c.378_379delTGinsAT | p.Asp126_Glu127delinsGluTer |
| *CHEK2* | c.409C>T | p.Arg137Ter |
| *CHEK2* | c.433C>T | p.Arg145Trp |
| *CHEK2* | c.444+1G>A |  |
| *CHEK2* | c.483_485delAGA | p.Glu161del |
| *CHEK2* | c.683+1G>T |  |
| *CHEK2* | c.836delA | p.Lys279Serfs*2 |
| *CHEK2* | c.860delA | p.Lys287Argfs*17 |
| *CHEK2* | c.917G>C | p.Gly306Ala |
| *CHEK2* | c.1100delC | p.Thr367Metfs*15 |
| *CHEK2* | c.1263delT | p.Ser422Valfs*15 |
| *CHEK2* | c.1368dupA | p.Glu457Argfs*33 |
| *CHEK2* | c.1370dupA | p.Lys458Glufs*32 |
| *CHEK2* | c.1427C>T | p.Thr476Met |
| *CHEK2* | c.1486C>T | p.Gln496Ter |
| *CHEK2* | c.1555C>T | p.Arg519Ter |
| *NBN* | c.657_661delACAAA | p.Lys219Asnfs*16 |
| *NBN* | c.873_874insA | p.Ser292Ilefs*12 |
| *NBN* | c.1142delC | p.Pro381Glnfs*23 |
| *NBN* | c.1903A>T | p.Lys635Ter |
| *NBN* | c.2071-1G>A |  |
| *PALB2* | c.196C>T | p.Gln66Ter |
| *PALB2* | c.212-2A>G |  |
| *PALB2* | c.424A>T | p.Lys142Ter |
| *PALB2* | c.509_510delGA | p.Arg170Ilefs*14 |
| *PALB2* | c.658delA | p.Ser220Valfs*3 |
| *PALB2* | c.758dupT | p.Ser254Ilefs*3 |
| *PALB2* | c.760dupT | p.Ser254Phefs*3 |
| *PALB2* | c.1240C>T | p.Arg414Ter |
| *PALB2* | c.1467_1468delTC | p.Pro490Argfs*5 |
| *PALB2* | c.1592delT | p.Leu531Cysfs*30 |
| *PALB2* | c.2167_2168delAT | p.Met723Valfs*21 |
| *PALB2* | c.2267_2283dup17 | p.His762Alafs*8 |
| *PALB2* | c.2323C>T | p.Gln775Ter |
| *PALB2* | c.2325dupA | p.Phe776Ilefs*26 |
| *PALB2* | c.2834+1G>A |  |
| *PALB2* | c.2976_2977insAT | p.Thr993Ilefs*15 |
| *PALB2* | c.3113G>A | p.Trp1038Ter |
| *PALB2* | c.3374_3395del22 | p.Asp1125Glyfs*31 |
| *PALB2* | c.3549C>G | p.Tyr1183Ter |
| *RAD51C* | c.93delG | p.Phe32Serfs*8 |
| *RAD51C* | c.97C>T | p.Gln33Ter |
| *RAD51C* | c.225delT | p.Tyr75Ter |
| *RAD51C* | c.397C>T | p.Gln133Ter |
| *RAD51C* | c.404G>C | p.Cys135Ser |
| *RAD51C* | c.414G>C | p.Leu138Phe |
| *RAD51C* | c.706-2A>G |  |
| *RAD51C* | c.709C>T | p.Arg237Ter |
| *RAD51C* | c.774delT | p.Thr259Leufs*4 |
| *RAD51C* | c.904+5G>T |  |
| *RAD51C* | c.905-2_905-1delAG |  |
| *RAD51C* | c.905-2A>G |  |
| *RAD51C* | c.955C>T | p.Arg319Ter |
| *RAD51C* | c.1026+5_1026+7delGTA |  |
| *RAD51D* | c.1A>G | p.Met1? |
| *RAD51D* | c.1A>T | p.Met1? |
| *RAD51D* | c.81delA | p.Val28Trpfs*12 |
| *RAD51D* | c.478C>T | p.Gln160Ter |
| *RAD51D* | c.556C>T | p.Arg186Ter |
| *RAD51D* | c.620C>T | p.Ser207Leu |
| *RAD51D* | c.694C>T | p.Arg232Ter |
| *RAD51D* | c.748delC | p.His250Thrfs*2 |
| *RAD51D* | c.772_778delGGGAGGC | p.Gly258Serfs*50 |
| *RAD51D* | c.803G>A | p.Trp268Ter |
| *RAD51D* | c.857delC | p.Ala286Aspfs*24 |
| *RAD51D* | c.898C>T | p.Arg300Ter |

RefSeq transcripts: *ATM* (NM_000051.3), *BARD1* (NM_000465.2), *BRCA1* (NM_007294.3), *BRCA2* (NM_000059.3), *BRIP1* (NM_032043.2), *CHEK2* (NM_007194.3), *NBN* (NM_002485.4), *PALB2* (NM_024675.3), *RAD51C* (NM_058216.1), *RAD51D* (NM_002878.3).
