# Supplementary material for: Age-adjusted association of homologous recombination genes with ovarian cancer using clinical exomes as controls
Source: Hered Cancer Clin Pract. 2019 Jul 15;17:19. doi: 10.1186/s13053-019-0119-3 (PMC6631909; doi:10.1186/s13053-019-0119-3)
Supplement: Supplementary file 4 — Calculation of the z-Statistic. The formula used to determine if crude odds ratios were significantly different than adjusted odds ratios. (DOCX 12 kb) [file 13053_2019_119_MOESM4_ESM.docx]

**Calculation of Z-statistic for Effect Size Comparisons**

Crude and adjusted odds ratios were compared by calculating a z statistic using the following equation:

$$z = \frac{\hat{\beta}_{Adjusted}-\hat{\beta}_{Crude}}{\sqrt{{SE\left( \hat{\beta}_{Adjusted} \right)}^{2}+{SE\left( \hat{\beta}_{Crude} \right)}^{2}}}$$

Where $\hat{\beta}$ is the parameter estimate for the beta coefficient for a pathogenic variant from the logistic regression model (expressed as log odds) and $SE(\hat{\beta)}$is the standard error of the coefficient parameter estimates. P-values for each gene were calculated by comparing *z* to a standard normal distribution.
