# Supplementary material for: Age-adjusted association of homologous recombination genes with ovarian cancer using clinical exomes as controls
Source: Hered Cancer Clin Pract. 2019 Jul 15;17:19. doi: 10.1186/s13053-019-0119-3 (PMC6631909; doi:10.1186/s13053-019-0119-3)
Supplement: Supplementary file 5 — Serous-subtype ovarian cancer sensitivity analysis. Results when limited to women with serous-subtype ovarian cancer. (DOCX 14 kb) [file 13053_2019_119_MOESM5_ESM.docx]

**Additional file 5: Table S4.** Associations of Serous-Subtype Ovarian Cancer with Pathogenic Variants in Homologous Recombination Genes among Self-Reported White Women.

| Gene | Median Age of PV Carriers (Cases^a^, Controls^b^) | Crude Odds Ratio (CI) | p-value, Crude^c^ | Adjusted Odds Ratio (CI) | p-value, Adjusted^c^ | Case Carriers, No. (Non-Carriers)^d^ | Control Carriers, No.  (Non-Carriers) | p-value, z-test^e^ |
| --- | --- | --- | --- | --- | --- | --- | --- | --- |
| *ATM* | 60.0, 47.0 | 1.31. (0.57 to 2.83) | 0.70 | 0.61 (0.21 to 1.66) | 0.57 | 9 (1939) | 17 (4673) | 0.14 |
| *BARD1* | 66.0, 34.5 | 1.66 (0.15 to 12.46) | 0.70 | 4.32 (0.06 to 70.12) | 0.77 | 1 (1698) | 2 (4688) | 0.70 |
| *BRCA1* | 54.0, 37.0 | 50.11 (21.93 to 151.24) | 5.12x10^-47^ | 87.66 (36.52 to 272.70) | 4.36x10^-47^ | 115 (2400) | 4 (4686) | 0.73 |
| *BRCA2* | 63.0, 40.0 | 11.16 (6.97 to 18.92) | 4.23x10^-31^ | 8.50 (4.77 to 15.87) | 1.61x10^-14^ | 106 (2409) | 18 (4672) | 0.23 |
| *BRIP1* | 65.0, 46.0 | 2.92 (1.47 to 5.91) | 4.74x10^-03^ | 1.25 (0.50 to 3.19) | 0.77 | 18 (2044) | 14 (4676) | 0.07 |
| *CHEK2* | 63.5, 42.0 | 1.09 (0.53 to 2.12) | 0.81 | 1.66 (0.63 to 4.02) | 0.57 | 12 (2006) | 24 (4287) | 0.77 |
| *NBN* | 60.5, 37.0 | 0.71 (0.13 to 2.49) | 0.70 | 0.93 (0.14 to 4.95) | 0.94 | 2 (1744) | 9 (4681) | 0.65 |
| *PALB2* | 66.0, 39.0 | 2.24 (0.79 to 6.30) | 0.21 | 1.52 (0.35 to 7.20) | 0.77 | 7 (2094) | 7 (4683) | 0.34 |
| *RAD51C* | 58.0, 39.0 | 12.92 (2.91 to 121.42) | 8.61x10^-04^ | 11.38 (1.84 to 131.62) | 0.02 | 8 (2056) | 1 (4689) | 0.38 |
| *RAD51D* | 59.0, 32.5 | 10.51 (3.10 to 54.13) | 2.11x10^-04^ | 25.28 (4.84 to 165.13) | 2.92x10^-04^ | 11 (2051) | 2 (4688) | 0.72 |

^a^Age (in years) at time of ovarian cancer diagnosis

^b^Age (in years) at time of testing

^c^Corrected for False Discovery Rate

^d^All genes were not tested for every sample

^e^Comparing crude odds ratio to adjusted odds ratio

CI = Confidence Interval

No. = Number
