# Supplementary material for: Age-adjusted association of homologous recombination genes with ovarian cancer using clinical exomes as controls
Source: Hered Cancer Clin Pract. 2019 Jul 15;17:19. doi: 10.1186/s13053-019-0119-3 (PMC6631909; doi:10.1186/s13053-019-0119-3)
Supplement: Supplementary file 6 — Comparison of pathogenic variant prevalence with Lilyquist et al. Results from Fisher’s Exact Test comparing prevalence of pathogenic variants in homologous recombination genes to Lilyquist et al. (DOCX 14 kb) [file 13053_2019_119_MOESM6_ESM.docx]

**Additional file 6: Table S5.** Comparison between observed prevalence of pathogenic variants and prevalence of pathogenic variants reported by Lilyquist et al. in cases and controls.

| **Cases** | | | | | |  |  | **Controls** |  |  |
| --- | --- | --- | --- | --- | --- | --- | --- | --- | --- | --- |
| **Gene** | **Arvai et al. Non-Carriers, No.** | **Arvai et al. PV Carriers, No. (Frequency as %)** | **Lilyquist et al. Non-Carriers, No.** | **Lilyquist et al. PV Carriers, No. (Frequency as %)^a^** | **p-value^b^** | **Arvai et al. Non-Carriers, No.** | **Arvai et al. PV Carriers, No. (Frequency, as %)** | **Lilyquist et al. Non-Carriers, No.^c^** | **Lilyquist et al. PV Carriers, No. (Frequency, as %)** | **p-value^b^** |
| *ATM* | 4724 | 35 (0.74) | 6261 | 54 (0.86) | 0.78 | 4673 | 17 (0.36) | 24905 | 95 (0.38) | >0.99 |
| *BARD1* | 4122 | 3 (0.07) | 6286 | 8 (0.13) | 0.78 | 4688 | 2 (0.04) | 24975 | 25 (0.10) | 0.60 |
| *BRCA1* | 5944 | 219 (3.55) | 7220 | 269 (3.59) | >0.99 | 4686 | 4 (0.09) | 24924 | 76 (0.30) | 0.05 |
| *BRCA2* | 5969 | 193 (3.13) | 7239 | 250 (3.34) | 0.78 | 4672 | 18 (0.38) | 24895 | 105 (0.42) | 0.89 |
| *BRIP1* | 4805 | 30 (0.62) | 6236 | 58 (0.92) | 0.28 | 4676 | 14 (0.30) | 24954 | 46 (0.18) | 0.28 |
| *CHEK2* | 4923 | 45 (0.91) | 6272 | 58 (0.92) | >0.99 | 4287 | 24 (0.56) | 24768 | 232 (0.93) | 0.07 |
| *NBN* | 4232 | 4 (0.09) | 6272 | 22 (0.35) | 0.09 | 4681 | 9 (0.19) | 24957 | 43 (0.17) | 0.88 |
| *PALB2* | 5004 | 16 (0.32) | 6315 | 22 (0.35) | >0.99 | 4683 | 7 (0.15) | 24972 | 28 (0.11) | 0.81 |
| *RAD51C* | 4809 | 23 (0.48) | 6250 | 44 (0.70) | 0.35 | 4689 | 1 (0.02) | 24966 | 34 (0.14) | 0.11 |
| *RAD51D* | 4809 | 20 (0.41) | 5732 | 11 (0.19) | 0.23 | 4688 | 2 (0.04) | 24993 | 7 (0.03) | 0.88 |

^a^Estimated by multiplying the reported case allele count by # of cases tested.

^b^From Fisher’s exact test, corrected for false discovery rate

^c^Estimated by multiplying the reported control allele frequency by 2 then multiplying by 25,000 (the estimated number of reported controls).

PV = Pathogenic variant

No. = Number
